# Explaining rural–urban disparities in child stunting in African least developed countries: a multi-country cross-sectional analysis

**DOI:** 10.1186/s12939-026-02898-9

**Published:** 2026-06-03

**Authors:** Zhixin Liu, Dongsheng Zhao, Junkai Lin, Xiali Chen

**Affiliations:** 1 School of Public Health, Peking University, Haidian District, Beijing, 100191 China; 2 Yuncheng Central Hospital Affiliated to Shanxi Medical University, Yuncheng, Shanxi 044000 China

**Keywords:** Rural–urban disparities, Stunting, Least developed countries, Health inequality, KHB decomposition

## Abstract

**Background:**

Rural–urban disparities in child stunting persist in African least developed countries (LDCs), undermining progress towards Sustainable Development Goal 2.2. However, multi-country evidence remains limited on which underlying factors account for these disparities. This study examined rural–urban differences in child stunting across 18 African LDCs and assessed the relative contributions of distal socioeconomic resources and proximal household environmental, healthcare-access, and dietary conditions.

**Methods:**

We used the most recent Demographic and Health Surveys conducted between 2015 and 2024 in 18 African LDCs. The analytic sample included 88,625 children aged 6–59 months. Child stunting was defined as length/height-for-age z-scores below − 2 standard deviations from the WHO Child Growth Standards median. Explanatory factors included household wealth, maternal education, maternal digital access, water, sanitation and hygiene (WASH) conditions, healthcare access, and dietary diversity. Logistic regression and Karlson–Holm–Breen decomposition models were fitted separately within each country to estimate the rural–urban association in child stunting and the extent to which explanatory factors accounted for this association. Log odds ratios and decomposition estimates were then pooled across countries using random-effects meta-analysis.

**Results:**

Stunting prevalence was higher among rural than urban children in all 18 African LDCs, with rural–urban differences ranging from 4.2 to 33.1 percentage points across countries. In the KHB decomposition analysis, the pooled total odds ratio for child stunting associated with rural residence was 1.904 (95% CI: 1.644–2.205). All explanatory factors jointly accounted for 70.2% of the pooled rural–urban association. Household wealth made the largest contribution, accounting for 29.7% of the total association, followed by maternal digital access (23.6%) and maternal education (9.2%). WASH conditions accounted for 5.7%, whereas healthcare access and dietary diversity made limited contributions.

**Conclusion:**

Rural–urban inequalities in child stunting remain widespread across African LDCs. These inequalities were largely accounted for by unequal socioeconomic resources, especially household wealth, maternal digital access, and maternal education, while proximal household environmental, healthcare-access, and dietary factors made smaller or limited contributions. Narrowing this gap requires multisectoral strategies that address socioeconomic disadvantage, digital exclusion, and basic infrastructure deficits.

**Supplementary Information:**

The online version contains supplementary material available at 10.1186/s12939-026-02898-9.

## Introduction

Ending all forms of child malnutrition is a central target of the Sustainable Development Goals (SDG 2.2). Although global progress has been made in reducing childhood stunting, current trends remain insufficient to achieve the 2030 target, and children in Africa are expected to account for a large proportion of those left behind [1]. This challenge is particularly acute in least developed countries (LDCs). The United Nations defines LDCs as countries facing severe structural impediments to sustainable development, characterized by low income, weak human assets, and high economic and environmental vulnerability [2]. These structural constraints are linked to the social, environmental, and service conditions that shape child growth, including poverty, maternal education, food environments, water and sanitation infrastructure, healthcare access, and access to information. Because child stunting has long-term consequences for cognitive development, educational attainment, adult productivity, and later-life chronic disease risk, persistent stunting in African LDCs represents both a public health challenge and a broader development priority [3, 4].

Rural–urban inequalities in child stunting remain widespread across low- and middle-income countries [5]. Only 15 of 52 countries (28.8%) showed reductions in these inequalities between 1990 and 2021 [6]. Rural children often experience a higher burden of stunting than urban children, reflecting unequal access to the resources and conditions that support healthy growth, including household wealth, maternal education, water, sanitation, and hygiene (WASH) conditions, healthcare access, diverse food environments, and health-related information [7–9]. Identifying which of these factors account for rural–urban differences in stunting is essential for designing policies that are both equity-oriented and context-specific.

The updated UNICEF framework on the determinants of maternal and child nutrition shifts focus toward protecting and promoting the diets, environments and services that support optimal nutrition, growth, and development [10]. While these are considered key modifiable determinants of child stunting, evidence from controlled trials assessing their impact has varied across settings. For example, a community-led sanitation intervention in Mali reported improvements in child growth [11], whereas large factorial trials in Kenya [12], Zimbabwe [13], and Bangladesh [14] evaluating household water treatment, sanitation, handwashing, and complementary feeding interventions found limited or inconsistent effects on linear growth. These differences may reflect variation in baseline environmental contamination, intervention adherence, implementation context, and socioeconomic conditions. Moreover, controlled trials evaluate specific intervention packages under defined study conditions, whereas public health decision-making also requires evidence from routine, real-world population settings to assess how risk factors and inequalities operate at the population level [15]. Nationally representative survey data can therefore provide complementary evidence on which household environmental, healthcare-access, dietary, and socioeconomic factors account for rural–urban disparities in child stunting.

Survey-based evidence from African LDCs on rural–urban disparities in child stunting remains limited, with existing decomposition studies mainly conducted in a small number of countries, including Malawi [16], Rwanda [17], and Ethiopia [18]. These studies consistently showed a rural disadvantage in childhood stunting and identified socioeconomic factors, such as household wealth and maternal education, as contributors to the rural–urban gap. However, they have focused on socioeconomic and demographic characteristics, with limited attention to the relative contribution of underlying household environmental, healthcare-access, and dietary factors. According to the UNICEF framework, feeding practices, healthcare access, and environmental conditions are key underlying determinants of child nutrition [10]. However, previous African LDC studies have rarely examined the relative contribution of these factors within a decomposition framework. In addition, maternal digital access has rarely been considered, although digital connectivity may reflect an increasingly relevant dimension of socioeconomic resources and access to communication and information channels [19]. Digital access is patterned by household wealth, maternal education, and place of residence, but it may also capture aspects of information access and service navigation not fully represented by conventional socioeconomic indicators [20, 21]. It therefore remains unclear whether rural–urban disparities in child stunting in African LDCs are mainly accounted for by upstream socioeconomic resources, by more proximal household environment, healthcare-access, and dietary factors, or by their combined contribution.

To address these gaps, this study used the most recent Demographic and Health Surveys (DHS) from 18 African LDCs to examine rural–urban disparities in child stunting and to compare the relative contribution of multiple measured explanatory factors. These explanatory factors were organized into distal socioeconomic resources, including household wealth, maternal education, and maternal digital access, and proximal WASH conditions, healthcare access, and dietary diversity. By comparing these contributions, this study provides evidence on which measured factors most strongly account for rural–urban inequalities in child stunting across African LDCs.

## Methods

### Study design and participants

This study used a multi-country, cross-sectional design and was reported in accordance with the Strengthening the Reporting of Observational Studies in Epidemiology (STROBE) guidelines. The DHS Program collects nationally representative data on population, health, and nutrition indicators using standardized questionnaires and a standardized two-stage cluster sampling design. Details of the DHS sampling procedures have been described elsewhere [22].

We included the most recent publicly available DHS conducted between 2015 and 2024 in Least Developed Countries (LDCs). According to the United Nations classification [2], inclusion in the LDC category is based on three specific thresholds: (1) Gross National Income (GNI) per capita of ≤$1,088; (2) a Human Assets Index (HAI) of ≤ 60, which incorporates health (including the prevalence of stunting) and education indicators; and (3) an Economic and Environmental Vulnerability Index (EVI) of ≥ 36. Surveys from 2015 onward were selected to reflect the Sustainable Development Goals era and to ensure that the analysis was based on relatively recent and comparable data on child nutrition, household living conditions, healthcare access, and maternal digital access. This selection resulted in 18 countries: Angola, Benin, Burkina Faso, Burundi, Ethiopia, the Gambia, Guinea, Lesotho, Liberia, Madagascar, Malawi, Mali, Mozambique, Rwanda, Senegal, Sierra Leone, Tanzania, and Zambia. Detailed characteristics of the included surveys are provided in Supplementary Table 1.

Eligible participants were children aged 6–59 months. We excluded children who had died before the survey and children with missing or implausible length/height-for-age z-scores, defined as values below − 6 or above + 6 standard deviations according to WHO criteria.

Ethical approval for each DHS was obtained from the ICF Institutional Review Board and the relevant national ethics review committee in each country. Informed consent was obtained during the original surveys. This study used publicly available, de-identified DHS data and did not require additional ethical approval.

### Variables

#### Outcome

The outcome was child stunting. Stunting was defined as a length/height-for-age z-score below − 2 standard deviations from the WHO Child Growth Standards median [23]. We used the z-scores provided in the DHS child recode files, which were calculated according to the WHO Child Growth Standards based on children’s anthropometric measurements, age, and sex. Anthropometric measurements were collected by trained field staff following standard survey protocols and with parental consent [22].

#### Exposure

The exposure of interest was place of residence, classified as urban or rural. This DHS classification was assigned at the survey-cluster or enumeration-area level using the official national definition in place at the time of data collection.

#### Explanatory variables

Explanatory factors were selected according to the UNICEF conceptual framework on maternal and child nutrition [10] and organized into distal and proximal domains.

Distal factors included household wealth, maternal education, and maternal digital access. Household wealth was measured using a harmonized asset-based index constructed from household economic assets and living-standard indicators that were consistently available across surveys, including ownership of durable goods, housing materials, land ownership, and livestock ownership. The resulting wealth score was divided into quintiles within each country, representing relative socioeconomic position rather than absolute cross-country wealth comparability. Maternal education was categorized as no education, primary education, and secondary or higher education. Maternal digital access served as a proxy for digital inclusion and informational connectivity, and was categorized into four levels based on mobile phone ownership and internet use frequency: (1) digitally excluded (no phone); (2) mobile phone only (no internet); (3) less frequent internet use; and (4) regular internet use (almost every day).

Proximal factors included WASH conditions, healthcare access barriers, and dietary diversity. WASH conditions were measured as the number of improved components available to the household, including improved drinking water, improved sanitation, and handwashing facilities, and were categorized from 0 to 3. Healthcare access barriers were measured using four consistently available DHS items: getting permission to seek care, getting money for treatment, distance to a health facility, and not wanting to go alone [24, 25]. We summed the number of reported barriers and treated the resulting score as a five-level categorical variable: 0, 1, 2, 3, or 4 barriers. Higher categories indicated a greater number of reported healthcare access barriers. The four-item measure showed acceptable internal consistency in the analytic sample (cronbach’s alpha = 0.72). Dietary diversity was constructed from DHS child feeding information based on reported food consumption in the previous 24 h. Food items were grouped into seven food groups: grains, roots, and tubers; legumes and nuts; dairy products; flesh foods; eggs; vitamin A-rich fruits and vegetables; and other fruits and vegetables. Children who consumed foods from four or more food groups were classified as meeting dietary diversity [26].

#### Covariates

We included child age in months, child sex, multiple birth status, birth order, and maternal age as covariates. Child age and maternal age were modelled as continuous variables. Child sex was categorized as male or female. Multiple birth status was coded as a binary variable, distinguishing singleton births from twin or higher-order multiple births. Birth order was categorized as first birth, second to fourth birth, and fifth or higher birth.

### Statistical analysis

Sample characteristics were summarized by place of residence. Categorical variables were presented as unweighted counts and percentages, and continuous variables as means and standard deviations. Differences between urban and rural children were assessed using Pearson chi-square tests for categorical variables and t tests for continuous variables. We estimated the prevalence of child stunting separately among urban and rural children in each country, accounting for sampling weights, primary sampling unit clustering, and stratification. Stunting prevalence was reported as percentages with 95% confidence intervals.

We examined the association between place of residence and child stunting using logistic regression models. Models were fitted separately for each country, accounting for DHS sampling weights, primary sampling unit clustering, and survey stratification. Log odds ratios and standard errors from individual country analyses were then pooled using random-effects meta-analysis. We fitted stepwise models by adjusting for demographic covariates, household wealth, maternal education, maternal digital access, improved WASH components, healthcare access barriers, and dietary diversity. Pooled estimates were exponentiated and reported as odds ratios with 95% confidence intervals.

We conducted Karlson–Holm–Breen (KHB) decomposition analyses to assess the extent to which prespecified explanatory factors accounted for the rural–urban difference in child stunting [27]. KHB models were fitted separately for each country using logistic regression, applying individual sampling weights and clustering standard errors at the primary sampling unit level. All models included child age, child sex, multiple birth status, birth order, and maternal age as concomitant covariates. Explanatory factors were evaluated in three prespecified KHB models: (1) included only distal factors, (2) included only proximal factors, and (3) included all measured explanatory factors. The procedure decomposed the rural–urban association into the total association, the direct association, and the indirect association through the explanatory factors, all estimated on the log odds-ratio scale. Log-scale estimates and standard errors were then pooled across countries using random-effects meta-analysis. The percentage accounted for was calculated as the pooled log-scale indirect association divided by the pooled total log odds ratio, multiplied by 100.

We conducted two sensitivity analyses. First, we repeated the main regression and KHB decomposition analyses among children aged 24–59 months, responding to the concern that stunting reflects a cumulative, longer-term growth process. Second, we repeated the analyses using complete-case data to assess whether the findings were sensitive to the use of multiple imputation.

Missing data were handled using multiple imputation by chained equations. Missingness was minimal for household wealth (*n* = 4, 0.0%) and moderate for the number of improved WASH components (*n* = 6,023, 6.8%), but substantial for dietary diversity (*n* = 35,524, 40.1%). We generated 20 imputed datasets. The imputation model included place of residence, demographic covariates, and all explanatory factors used in the analysis. Binary variables were imputed using logistic regression models, and ordered categorical variables were imputed using ordered logistic regression models. All analyses were conducted using the multiply imputed datasets, and estimates were combined according to Rubin’s rules.

All analyses were conducted using Stata/MP 17.0. A two-sided alpha level of 0.05 was considered statistically significant.

## Results

**Fig. 1:**
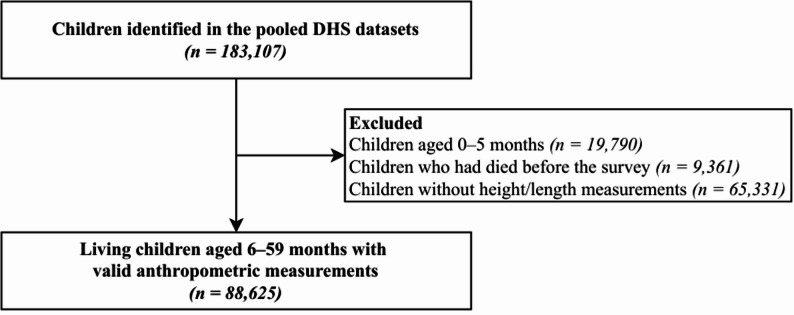
Flow diagram of the analytic sample selection

The analysis was restricted to living children aged 6–59 months with valid anthropometric measurements. We excluded children aged 0–5 months (*n* = 19,790), children who had died before the survey (*n* = 9,361), and children without height/length measurements required to define stunting (*n* = 65,331). The final analytic sample included 88,625 children (Fig. 1).

### Sample characteristics

Of the 88,625 children included in the analysis, 25,781 lived in urban areas and 62,844 in rural areas (Table 1). Child age and sex distributions were similar between urban and rural areas. Rural children were more likely than urban children to be fifth or higher birth order (32.7% vs. 20.9%).

**Table 1:** Characteristics of the study sample, stratified by urban and rural residence

Characteristic	Overall, *n* (%)	Type of residence		*P* value
		Urban, *n* (%)	Rural, *n* (%)	
**Child sex**				0.800
Male	44,850 (50.6)	13,064 (50.7)	31,786 (50.6)	
Female	43,775 (49.4)	12,717 (49.3)	31,058 (49.4)	
**Child age (months)**	31.38 (15.60)	31.27 (15.58)	31.42 (15.61)	0.184
**Birth order**				< 0.001
First birth	19,335 (21.8)	6,830 (26.5)	12,505 (19.9)	
Second to fourth birth	43,351 (48.9)	13,556 (52.6)	29,795 (47.4)	
Fifth or higher birth	25,939 (29.3)	5,395 (20.9)	20,544 (32.7)	
**Twin birth**				0.001
No	85,976 (97.0)	24,935 (96.7)	61,041 (97.1)	
Yes	2,649 (3.0)	846 (3.3)	1,803 (2.9)	
**Maternal age (years)**	29.43 (6.98)	29.31 (6.63)	29.48 (7.12)	< 0.001
**Household wealth**				< 0.001
Poorest	24,846 (28.0)	2,324 (9.0)	22,522 (35.8)	
Poorer	18,977 (21.4)	2,799 (10.9)	16,178 (25.7)	
Middle	16,178 (18.3)	5,146 (20.0)	11,032 (17.6)	
Richer	15,481 (17.5)	6,625 (25.7)	8,856 (14.1)	
Richest	13,139 (14.8)	8,885 (34.5)	4,254 (6.8)	
Missing	4 (0.0)	2 (0.0)	2 (0.0)	
**Maternal education**				< 0.001
No education	40,855 (46.1)	8,148 (31.6)	32,707 (52.0)	
Primary	28,712 (32.4)	7,114 (27.6)	21,598 (34.4)	
Secondary or higher	19,058 (21.5)	10,519 (40.8)	8,539 (13.6)	
**Maternal digital access**				< 0.001
Digitally excluded	48,323 (54.5)	7,691 (29.8)	40,632 (64.7)	
Mobile phone only	31,925 (36.0)	12,403 (48.1)	19,522 (31.1)	
Less frequent internet use	3,933 (4.4)	2,457 (9.5)	1,476 (2.3)	
Regular internet use	4,444 (5.0)	3,230 (12.5)	1,214 (1.9)	
**Number of improved WASH components**				< 0.001
0	20,000 (22.6)	2,090 (8.1)	17,910 (28.5)	
1	38,938 (43.9)	11,525 (44.7)	27,413 (43.6)	
2	19,717 (22.2)	7,858 (30.5)	11,859 (18.9)	
3	3,947 (4.5)	2,354 (9.1)	1,593 (2.5)	
Missing	6,023 (6.8)	1,954 (7.6)	4,069 (6.5)	
**Problems in accessing healthcare**				< 0.001
0	33,711 (38.0)	13,541 (52.5)	20,170 (32.1)	
1	20,401 (23.0)	5,952 (23.1)	14,449 (23.0)	
2	16,032 (18.1)	3,111 (12.1)	12,921 (20.6)	
3	10,452 (11.8)	1,749 (6.8)	8,703 (13.8)	
4	8,029 (9.1)	1,428 (5.5)	6,601 (10.5)	
**Dietary diversity**				< 0.001
No	43,194 (48.7)	10,760 (41.7)	32,434 (51.6)	
Yes	9,907 (11.2)	3,918 (15.2)	5,989 (9.5)	
Missing	35,524 (40.1)	11,103 (43.1)	24,421 (38.9)	

Rural–urban differences were observed in socioeconomic and informational characteristics. Compared with urban children, rural children were more often from the poorest households (35.8% vs. 9.0%), had mothers with no education (52.0% vs. 31.6%), and had digitally excluded mothers (64.7% vs. 29.8%). Regular maternal internet use was much less common in rural than urban areas (1.9% vs. 12.5%).

Rural children also had less favourable household and service conditions. They were more likely to have no improved WASH components (28.5% vs. 8.1%) and to report multiple healthcare access problems. Dietary diversity was met by 9.5% of rural children and 15.2% of urban children.

### Rural–urban disparities in stunting prevalence

**Fig. 2:**
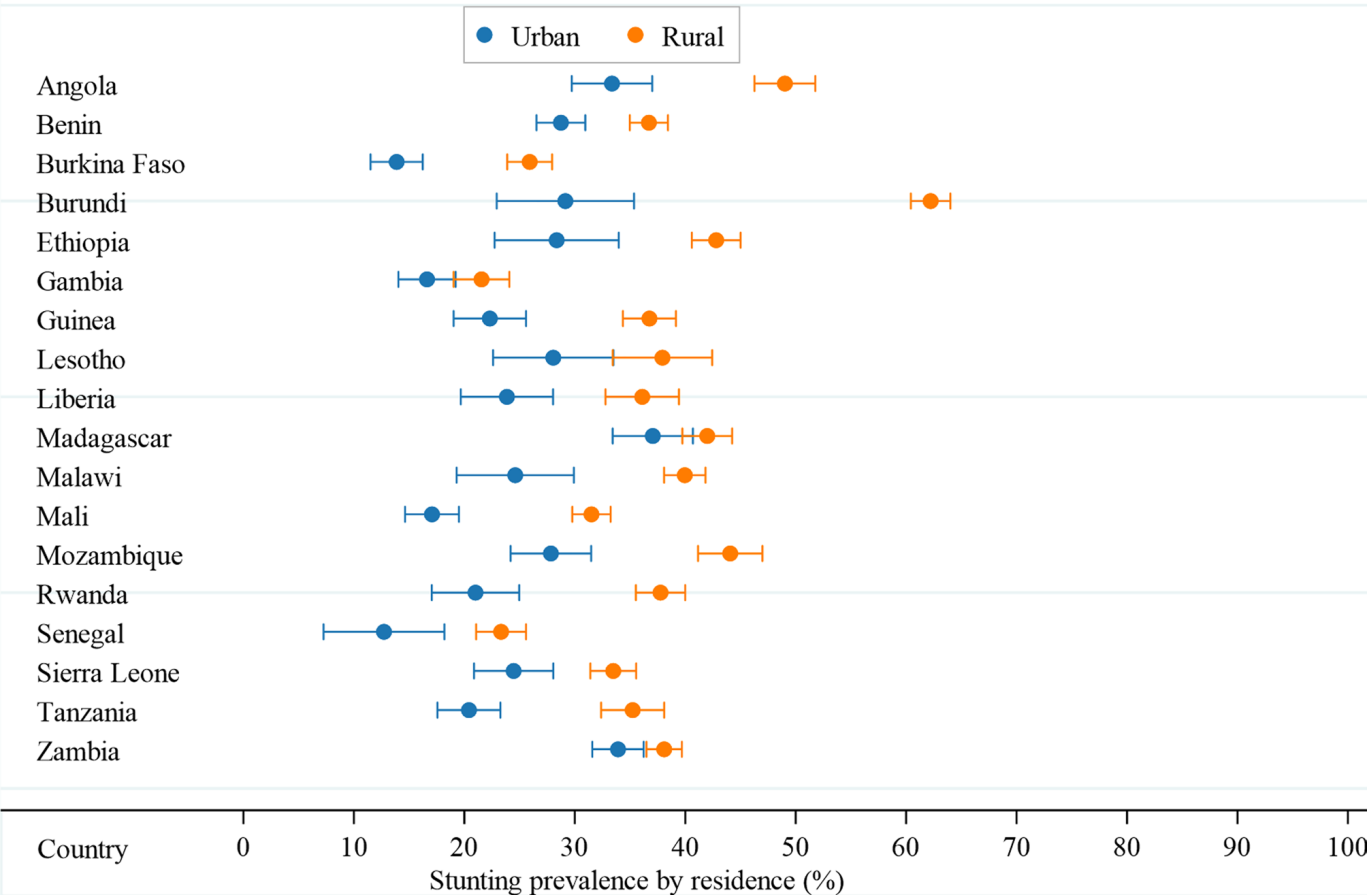
Prevalence of child stunting by place of residence across 18 African least developed countries. Notes: Points indicate weighted prevalence estimates, and horizontal bars indicate 95% confidence intervals

Rural children had a higher prevalence of stunting than urban children. Urban prevalence ranged from 12.7% in Senegal to 37.1% in Madagascar, while rural prevalence ranged from 21.6% in the Gambia to 62.2% in Burundi (Fig. 2). The rural–urban absolute difference ranged from 4.2 percentage points in Zambia to 33.1 percentage points in Burundi. The largest rural–urban gaps were observed in Burundi, Rwanda, Mozambique, Angola, Malawi, and Tanzania, whereas smaller gaps were observed in Zambia, Gambia, and Madagascar.

### Association between place of residence and child stunting

Rural residence was associated with higher odds of stunting than urban residence (pooled crude OR: 1.844; 95% CI: 1.614–2.107; Table 2). After adjustment for demographic covariates, rural residence remained associated with higher odds of stunting (pooled OR: 1.799; 95% CI: 1.571–2.060). The pooled OR decreased to 1.325 (95% CI: 1.182–1.486) after adjustment for household wealth, 1.264 (95% CI: 1.131–1.412) after adjustment for maternal education, and 1.194 (95% CI: 1.071–1.332) after adjustment for maternal digital access. Additional adjustment for WASH conditions, healthcare access barriers, and dietary diversity resulted in smaller changes. In the fully adjusted model, rural residence remained associated with higher odds of stunting (pooled OR: 1.171; 95% CI: 1.055–1.299).

**Table 2:** Pooled adjusted odds ratios for child stunting from logistic regression models

Variable	Crude OR (95% CI)	Model 1	Model 2	Model 3	Model 4	Model 5	Model 6	Model 7
		Adjusted for child age, child sex, multiple birth status, birth order, and maternal age	Model 1 + household wealth	Model 2 + maternal education	Model 3 + maternal digital access	Model 4 + WASH conditions	Model 5 + healthcare access barriers	Model 6 + dietary diversity
**Residence**								
Urban	1.000	1.000	1.000	1.000	1.000	1.000	1.000	1.000
Rural	1.844 (1.614–2.107)	1.799 (1.571–2.060)	1.325 (1.182–1.486)	1.264 (1.131–1.412)	1.194 (1.071–1.332)	1.178 (1.056–1.313)	1.171 (1.055–1.300)	1.171 (1.055–1.299)
**Household wealth**								
Poorest	1.000		1.000	1.000	1.000	1.000	1.000	1.000
Poorer	0.840 (0.794–0.888)		0.849 (0.804–0.896)	0.861 (0.816–0.909)	0.875 (0.831–0.921)	0.880 (0.836–0.926)	0.885 (0.841–0.932)	0.885 (0.840–0.931)
Middle	0.700 (0.636–0.770)		0.745 (0.680–0.816)	0.767 (0.702–0.839)	0.798 (0.736–0.866)	0.809 (0.744–0.880)	0.818 (0.752–0.889)	0.818 (0.753–0.890)
Richer	0.557 (0.499–0.622)		0.612 (0.547–0.686)	0.645 (0.577–0.720)	0.694 (0.623–0.773)	0.714 (0.642–0.795)	0.721 (0.646–0.803)	0.721 (0.646–0.803)
Richest	0.323 (0.264–0.395)		0.398 (0.327–0.484)	0.445 (0.370–0.534)	0.526 (0.445–0.622)	0.553 (0.468–0.655)	0.559 (0.472–0.663)	0.559 (0.471–0.662)
**Maternal education**								
No education	1.000			1.000	1.000	1.000	1.000	1.000
Primary	0.816 (0.736–0.903)			0.892 (0.813–0.978)	0.918 (0.838–1.006)	0.923 (0.844–1.010)	0.927 (0.847–1.015)	0.927 (0.848–1.014)
Secondary or higher	0.483 (0.402–0.579)			0.686 (0.602–0.781)	0.782 (0.690–0.887)	0.795 (0.701–0.902)	0.800 (0.705–0.908)	0.800 (0.706–0.907)
**Maternal digital access**								
Digitally excluded	1.000				1.000	1.000	1.000	1.000
Mobile phone only	0.621 (0.568–0.678)				0.772 (0.730–0.816)	0.776 (0.736–0.818)	0.779 (0.740–0.820)	0.778 (0.739–0.820)
Less frequent internet use	0.346 (0.271–0.442)				0.594 (0.503–0.702)	0.608 (0.519–0.712)	0.613 (0.524–0.716)	0.612 (0.525–0.714)
Regular internet use	0.229 (0.176–0.299)				0.451 (0.359–0.567)	0.474 (0.380–0.592)	0.481 (0.385–0.601)	0.480 (0.385–0.599)
**Number of improved WASH components**								
0	1.000					1.000	1.000	1.000
1	0.801 (0.751–0.855)					0.946 (0.902–0.993)	0.949 (0.904–0.995)	0.949 (0.904–0.995)
2	0.599 (0.539–0.664)					0.858 (0.803–0.916)	0.865 (0.810–0.923)	0.864 (0.809–0.922)
3	0.351 (0.293–0.421)					0.744 (0.656–0.844)	0.753 (0.662–0.856)	0.753 (0.664–0.855)
**Problems in accessing healthcare**								
0	1.000						1.000	1.000
1	1.194 (1.115–1.278)						1.036 (0.985–1.089)	1.037 (0.986–1.090)
2	1.332 (1.205–1.473)						1.037 (0.952–1.130)	1.038 (0.953–1.131)
3	1.442 (1.324–1.570)						1.096 (1.019–1.179)	1.097 (1.019–1.180)
4	1.524 (1.386–1.677)						1.153 (1.038–1.281)	1.141 (1.035–1.257)
**Dietary diversity**								
No	1.000							1.000
Yes	0.830 (0.772–0.893)							1.016 (0.958–1.077)

Notes: Values are pooled odds ratios (ORs) with 95% confidence intervals from logistic regression models. Models were fitted separately within each country, and country-level log-odds estimates were pooled using random-effects meta-analysis. Abbreviations: CI, confidence interval; OR, odds ratio; WASH, water, sanitation, and hygiene

In the fully adjusted model, higher household wealth, higher maternal education, greater maternal digital access, and more improved WASH components were associated with lower odds of stunting (Table 2). Compared with children from the poorest households, children from the richest households had lower odds of stunting (pooled OR: 0.559; 95% CI: 0.471–0.662). Compared with children whose mothers had no education, children whose mothers had secondary or higher education had lower odds of stunting (pooled OR: 0.800; 95% CI: 0.706–0.907). Compared with children living in households with no improved WASH components, those living in households with three improved WASH components had lower odds of stunting (pooled OR: 0.753; 95% CI: 0.664–0.855). Compared with children whose mothers reported no healthcare access barriers, those whose mothers reported four barriers had higher odds of stunting (pooled OR: 1.141; 95% CI: 1.035–1.257). Dietary diversity was not associated with stunting after full adjustment (pooled OR: 1.016; 95% CI: 0.958–1.077).

### KHB decomposition analyses

Table 3 presents the pooled KHB decomposition results for the rural–urban difference in child stunting. In Panel A, distal and proximal explanatory domains were examined separately. In the model including only distal socioeconomic resources, household wealth made the largest contribution, explaining 33.2% of the total rural–urban association, followed by maternal digital access (25.0%) and maternal education (10.1%). In the model including proximal household, care, and dietary conditions, improved WASH components explained 16.3%, problems in accessing healthcare explained 5.0%, and dietary diversity explained 0.2%.

**Table 3:** Pooled KHB decomposition of the rural–urban difference in child stunting across 18 African least developed countries

	Pooled point estimate	95% confidence interval	% of total rural–urban association explained
**Panel A. Explanatory factor groups examined separately**			
**Distal socioeconomic resources**			
**Total association**	1.895	1.636–2.195	NA
**Indirect association of each explanatory factor**			
Household wealth	1.236	1.167–1.309	33.2
Maternal education	1.067	1.035–1.100	10.1
Maternal digital access	1.174	1.132–1.217	25.0
**Proximal household**,** care**,** and dietary conditions**			
**Total association**	1.825	1.589–2.096	NA
**Indirect association of each explanatory factor**			
Improved WASH components	1.103	1.073–1.133	16.3
Problems in accessing healthcare	1.031	1.020–1.042	5.0
Dietary diversity	1.002	0.998–1.005	0.2
**Panel B. Full decomposition model**			
**All measured explanatory factors**			
**Total association**	1.904	1.644–2.205	NA
**Indirect association of each explanatory factor**			
Household wealth	1.211	1.142–1.284	29.7
Maternal education	1.061	1.029–1.094	9.2
Maternal digital access	1.164	1.123–1.207	23.6
Improved WASH components	1.038	1.022–1.054	5.7
Problems in accessing healthcare	1.013	1.003–1.023	2.0
Dietary diversity	1.000	0.997–1.003	0.0

Notes: KHB decomposition was performed separately for each country using logistic regression models on the log-odds scale. Country-level KHB estimates were then pooled using random-effects meta-analysis. The pooled point estimates shown in the table are exponentiated pooled log-scale KHB estimates. The percentage of the total rural–urban association explained was calculated on the log-odds scale. For individual explanatory factors, it was calculated as the pooled log-scale contribution of that factor divided by the pooled log-scale total rural–urban association in the corresponding model, multiplied by 100. Panel A shows models in which the two groups of explanatory factors were examined separately: distal socioeconomic resources and proximal household, care, and dietary conditions. Panel B shows the full decomposition model in which all measured explanatory factors were examined together. Abbreviations: KHB, Karlson–Holm–Breen; WASH, water, sanitation, and hygiene; NA, not available

Panel B presents the model including all measured explanatory factors. Household wealth remained the largest contributor, explaining 29.7% of the total rural–urban association. Maternal digital access explained 23.6%, and maternal education explained 9.2%. Improved WASH components and problems in accessing healthcare made smaller contributions, explaining 5.7% and 2.0%, respectively. Dietary diversity made almost no contribution.

### Sensitivity analyses

In the analysis restricted to children aged 24–59 months, the results were similar to the main analysis (Supplementary Tables 2 and 3). Rural residence was associated with higher odds of stunting in the crude model (OR: 1.919; 95% CI: 1.653–2.227). This association decreased after sequential adjustment and remained statistically significant in the full model (OR: 1.149; 95% CI: 1.021–1.292). The KHB results also showed a similar pattern. In the full decomposition model, household wealth explained the largest proportion of the rural–urban association (28.2%), followed by maternal digital access (24.7%) and maternal education (9.3%). WASH conditions explained 6.7%, while healthcare access barriers and dietary diversity made small or minimal contributions.

In the complete-case analysis, the findings were also broadly consistent with the main results (Supplementary Tables 4 and 5). Rural residence was associated with higher odds of stunting in the crude model (OR: 1.777; 95% CI: 1.542–2.048), and the association remained after full adjustment (OR: 1.142; 95% CI: 1.013–1.288). In the full decomposition model, household wealth again made the largest contribution (34.4%), followed by maternal digital access (22.1%) and maternal education (6.9%). WASH conditions explained 7.4%, healthcare access barriers explained 2.1%, and dietary diversity made almost no contribution. Overall, both sensitivity analyses supported the robustness of the main findings.

## Discussion

Using recent DHS data from 18 African LDCs, this study examined rural–urban disparities in child stunting and assessed the extent to which these disparities were accounted for by household wealth, maternal education, maternal digital access, WASH conditions, healthcare access, and dietary diversity. Several findings emerged. First, stunting prevalence was higher among rural than urban children in all included countries, although the magnitude of the rural–urban gap varied substantially. Second, child stunting was associated with lower household wealth, lower maternal education, lower maternal digital access, fewer improved WASH components, and more healthcare access barriers. Third, the KHB decomposition shows that the measured explanatory factors jointly accounted for 70.2% of the total rural–urban association.

The rural disadvantage observed in our study is consistent with recent evidence from 52 low- and middle-income countries [6]. Our decomposition analysis showed that household wealth was the largest contributor to the rural–urban stunting difference, while maternal education also contributed to the gap. This is broadly consistent with decomposition studies from African LDCs, including Malawi [16], Rwanda [17], and Ethiopia [18], which reported that rural-urban differences in child stunting were partly attributable to socioeconomic and demographic factors. Our study extends this evidence by assessing the relative contribution of proximal household environment, healthcare-access, and dietary factors. Our finding that WASH conditions explained part of the rural–urban difference is consistent with evidence from China, whereas early adequate feeding and healthcare access contributed little [28]. Maternal digital access also accounted for part of the rural–urban difference, suggesting that informational connectivity may represent an additional dimension of rural–urban inequality that has not been explicitly examined in previous stunting decomposition studies.

The larger contribution of distal socioeconomic resources, particularly household wealth, suggests that relative socioeconomic position remains an important axis of rural–urban inequality in child stunting, even within the constrained economic context of African LDCs [29]. Maternal education also contributed to the rural–urban difference, consistent with its established role in shaping health knowledge, care practices, service use, and household decision-making [30]. Its smaller contribution when household wealth and maternal digital access were included in the same model may reflect overlap among these socially patterned resources. Maternal digital access should also be interpreted within this broader socioeconomic context. In the regression models, adjustment for maternal digital access further attenuated the rural–urban odds ratio after household wealth and maternal education had already been included, while the associations for household wealth and maternal education were also modestly reduced. This suggests that maternal digital access partly overlaps with conventional socioeconomic position, but may also capture aspects of communication and information access not fully represented by household wealth or maternal education. However, because DHS does not measure direct use of digital health services, maternal mobile phone ownership and internet-use frequency should be understood as indicators of basic digital connectivity rather than digital health engagement. These findings should not be interpreted as evidence that digital access alone can reduce childhood stunting. Rather, digital connectivity may form part of a broader set of socioeconomic and informational resources that shape mothers’ ability to obtain childcare and nutrition information, communicate with social networks, and navigate health and social services, particularly in rural settings [19]. At the same time, digital health equity research cautions that digital technologies may reproduce or widen health inequalities when access, skills, affordability, and usability are unequally distributed [31, 32]. Future research should examine the specific pathways through which maternal digital access may influence child health, including health information seeking, social support, service navigation, care-seeking behaviour, or other mechanisms.

WASH conditions, healthcare access barriers, and dietary diversity accounted for part of the rural–urban difference, but their additional statistical contributions were smaller when household wealth, maternal education, and maternal digital access were considered simultaneously. This suggests that rural–urban differences in WASH, healthcare access, and dietary diversity may be embedded in broader socioeconomic disadvantage [10]. Households with fewer economic resources may have poorer access to improved water and sanitation facilities, face greater barriers in reaching healthcare services, and have more limited capacity to provide diverse foods for young children [33, 34]. Similarly, maternal education and digital access may shape health knowledge, care-seeking, service navigation, and feeding-related information [35–38]. Therefore, the smaller additional contribution of these proximal factors may reflect overlap between upstream socioeconomic resources and downstream household environmental, service, and dietary conditions.

The findings have several policy implications. First, they show that rural–urban inequalities in child stunting remain substantial across African LDCs, with particularly large rural disadvantages observed in countries such as Burundi, Rwanda, Mozambique, Angola, Malawi, and Tanzania. These disparities suggest that reducing child stunting in African LDCs will require attention to rural disadvantage, rather than only overall national reductions in stunting prevalence. Second, our regression analyses showed that child stunting was associated with household wealth, maternal education, maternal digital access, WASH conditions, and healthcare access barriers. The decomposition results further suggested that household wealth and maternal digital access made the largest contributions to the rural–urban stunting gap, followed by maternal education, while WASH conditions and healthcare access contributed more modestly after these upstream factors were considered. These findings suggest that broader rural development strategies, including poverty reduction, improvements in girls’ and women’s education, and efforts to reduce digital divide, may deserve greater policy attention alongside conventional nutrition, WASH, and healthcare strategies [39]. Further intervention and policy studies are needed to determine whether targeting these areas can reduce rural–urban inequalities in childhood stunting.

To our knowledge, this is the first multi-country study in African LDCs to compare the relative contribution of distal socioeconomic resources and proximal household environment, healthcare-access, and dietary factors to rural–urban disparities in child stunting. Several limitations should be acknowledged. First, this study used a cross-sectional design, and the KHB results should therefore be interpreted as decomposition estimates rather than evidence of causal mediation. Second, maternal digital access was treated as a distal socioeconomic resource related to digital connectivity. It was measured using mobile phone ownership and frequency of internet use, which capture basic digital connection but do not indicate whether mothers used digital tools for health or nutrition purposes. We also did not measure digital literacy, health literacy, engagement with digital health services, affordability or quality of internet access, or the relevance and reliability of online content. Therefore, we could not determine whether the contribution of maternal digital access reflected health information seeking, communication with social networks, service navigation, broader socioeconomic advantage, or a combination of these pathways. Third, dietary diversity was based on recent reported food consumption and may be affected by recall or reporting bias. It also had substantial missingness and may not fully capture long-term dietary adequacy, food security, micronutrient intake, or cumulative nutritional deprivation. Finally, unmeasured factors, such as food prices, health service quality, local infrastructure, environmental shocks, conflict, programme coverage, and governance, may still explain part of the remaining rural–urban difference [40, 41].

## Conclusion

Using recent DHS data collected between 2015 and 2024 from 18 African LDCs, this study found consistent rural disadvantages in child stunting, although the magnitude of the rural–urban disparity varied across countries. The measured explanatory factors jointly accounted for 70.2% of the pooled rural–urban association. Distal socioeconomic resources, particularly household wealth, maternal digital access, and maternal education, made the largest contributions, whereas WASH conditions contributed to a smaller extent and healthcare access barriers and dietary diversity contributed little. These findings suggest that rural–urban inequalities in childhood stunting in African LDCs are closely linked to unequal socioeconomic resources, digital connectivity, and household environmental conditions. Future research should assess whether interventions addressing these domains can reduce rural–urban inequalities in child stunting.

## Supplementary Information

Below is the link to the electronic supplementary material.

Supplementary Material 1

## Data Availability

The data used in this study are publicly available and can be obtained upon request to Demographic and Health Surveys (https://www.dhsprogram.com/).

## Abbreviations

CI: Confidence interval
DHS: Demographic and Health Surveys
EVI: Economic and environmental vulnerability index
GNI: Gross national income
HAI: Human assets index
KHB: Karlson–Holm–Breen
LDC: Least developed countries
OR: Odds ratio
SDG: Sustainable development goal
STROBE: Strengthening the reporting of observational studies in epidemiology
UNICEF: United Nations Children’s Fund
WASH: Water, sanitation and hygiene
WHO: World Health Organization
