# Supplementary material for: Explaining rural–urban disparities in child stunting in African least developed countries: a multi-country cross-sectional analysis
Source: Int J Equity Health. 2026 Jun 3;25:160. doi: 10.1186/s12939-026-02898-9 (PMC13321889; doi:10.1186/s12939-026-02898-9)
Supplement: Supplementary file 1 — Supplementary Material 1 [file 12939_2026_2898_MOESM1_ESM.docx]

**Supplementary Table 1. Characteristics of the 18 Least Developed Countries in Africa**

| **Country** | **Geographical subregion** | **Survey time** | **Sample size** |
| --- | --- | --- | --- |
| Angola | Middle Africa | 2015-16 | 5,571 |
| Benin | Western Africa | 2017-18 | 10,292 |
| Burkina Faso | Western Africa | 2021 | 5,108 |
| Burundi | Eastern Africa | 2016-17 | 5,426 |
| Ethiopia | Eastern Africa | 2016 | 7,903 |
| Gambia | Western Africa | 2019-20 | 3,278 |
| Guinea | Western Africa | 2018 | 3,036 |
| Lesotho | Southern Africa | 2023-24 | 956 |
| Liberia | Western Africa | 2019-20 | 2,170 |
| Madagascar | Eastern Africa | 2021 | 5,091 |
| Malawi | Eastern Africa | 2015-16 | 4,648 |
| Mali | Western Africa | 2018 | 7,351 |
| Mozambique | Eastern Africa | 2022-23 | 3,277 |
| Rwanda | Eastern Africa | 2019-20 | 3,435 |
| Senegal | Western Africa | 2023 | 5,458 |
| Sierra Leone | Western Africa | 2019 | 3,610 |
| Tanzania | Eastern Africa | 2022 | 4,254 |
| Zambia | Eastern Africa | 2018 | 7,761 |

**Note:** The geographical subregions are based on the United Nations Statistics Division (UNSD) geographical region classification

| **Supplementary Table 2. Pooled odds ratios for childhood stunting from logistic regression models: sensitivity analysis restricted to children aged 24–59 months** | | | | | | | | |
| --- | --- | --- | --- | --- | --- | --- | --- | --- |
|  | **Crude model** | **Model 1** | **Model 2** | **Model 3** | **Model 4** | **Model 5** | **Model 6** | **Model 7** |
| **Variable** |  | **Adjusted for child age, child sex, multiple birth status, birth order, and maternal age** | **Model 1 + household wealth** | **Model 2 + maternal education** | **Model 3 + maternal digital access** | **Model 4 + WASH conditions** | **Model 5 + healthcare access barriers** | **Model 6 + dietary diversity** |
| **Residence** |  |  |  |  |  |  |  |  |
| Urban | 1.000 | 1.000 | 1.000 | 1.000 | 1.000 | 1.000 | 1.000 | 1.000 |
| Rural | 1.919 (1.653–2.227) | 1.853 (1.596–2.151) | 1.330 (1.171–1.511) | 1.259 (1.112–1.426) | 1.176 (1.041–1.329) | 1.156 (1.024–1.305) | 1.149 (1.021–1.292) | 1.149 (1.021–1.292) |
| **Household wealth** |  |  |  |  |  |  |  |  |
| Poorest | 1.000 |  | 1.000 | 1.000 | 1.000 | 1.000 | 1.000 | 1.000 |
| Poorer | 0.803 (0.754–0.856) |  | 0.811 (0.760–0.866) | 0.823 (0.770–0.879) | 0.837 (0.785–0.892) | 0.843 (0.790–0.899) | 0.847 (0.794–0.903) | 0.847 (0.794–0.903) |
| Middle | 0.673 (0.606–0.748) |  | 0.721 (0.656–0.793) | 0.744 (0.680–0.815) | 0.778 (0.716–0.846) | 0.788 (0.723–0.859) | 0.795 (0.730–0.867) | 0.796 (0.731–0.867) |
| Richer | 0.516 (0.457–0.582) |  | 0.569 (0.507–0.638) | 0.604 (0.540–0.674) | 0.657 (0.590–0.731) | 0.679 (0.608–0.759) | 0.686 (0.614–0.766) | 0.686 (0.614–0.767) |
| Richest | 0.296 (0.238–0.368) |  | 0.365 (0.298–0.447) | 0.415 (0.344–0.501) | 0.497 (0.421–0.588) | 0.528 (0.443–0.630) | 0.534 (0.448–0.636) | 0.534 (0.448–0.636) |
| **Maternal education** |  |  |  |  |  |  |  |  |
| No education | 1.000 |  |  | 1.000 | 1.000 | 1.000 | 1.000 | 1.000 |
| Primary | 0.836 (0.756–0.924) |  |  | 0.911 (0.827–1.004) | 0.944 (0.858–1.039) | 0.951 (0.865–1.045) | 0.953 (0.868–1.047) | 0.954 (0.869–1.047) |
| Secondary or higher | 0.454 (0.372–0.554) |  |  | 0.659 (0.567–0.767) | 0.773 (0.670–0.891) | 0.789 (0.684–0.911) | 0.792 (0.686–0.914) | 0.793 (0.688–0.914) |
| **Maternal digital access** |  |  |  |  |  |  |  |  |
| Digitally excluded | 1.000 |  |  |  | 1.000 | 1.000 | 1.000 | 1.000 |
| Mobile phone only | 0.562 (0.512–0.617) |  |  |  | 0.722 (0.683–0.764) | 0.728 (0.688–0.770) | 0.731 (0.692–0.774) | 0.731 (0.691–0.773) |
| Less frequent internet use | 0.315 (0.241–0.411) |  |  |  | 0.579 (0.483–0.693) | 0.594 (0.502–0.702) | 0.597 (0.505–0.706) | 0.597 (0.505–0.706) |
| Regular internet use | 0.190 (0.136–0.264) |  |  |  | 0.410 (0.305–0.550) | 0.436 (0.323–0.588) | 0.443 (0.328–0.598) | 0.442 (0.328–0.596) |
| **Number of improved WASH facilities** |  |  |  |  |  |  |  |  |
| 0 | 1.000 |  |  |  |  | 1.000 | 1.000 | 1.000 |
| 1 | 0.787 (0.725–0.854) |  |  |  |  | 0.937 (0.868–1.011) | 0.940 (0.872–1.013) | 0.940 (0.873–1.013) |
| 2 | 0.561 (0.498–0.632) |  |  |  |  | 0.835 (0.767–0.909) | 0.841 (0.773–0.916) | 0.841 (0.773–0.914) |
| 3 | 0.318 (0.258–0.391) |  |  |  |  | 0.680 (0.558–0.830) | 0.688 (0.563–0.841) | 0.689 (0.565–0.841) |
| **Problems in accessing healthcare** |  |  |  |  |  |  |  |  |
| 0 | 1.000 |  |  |  |  |  | 1.000 | 1.000 |
| 1 | 1.221 (1.137–1.311) |  |  |  |  |  | 1.049 (0.986–1.116) | 1.049 (0.986–1.116) |
| 2 | 1.373 (1.225–1.538) |  |  |  |  |  | 1.040 (0.939–1.151) | 1.040 (0.939–1.151) |
| 3 | 1.494 (1.362–1.638) |  |  |  |  |  | 1.112 (1.022–1.210) | 1.112 (1.021–1.210) |
| 4 | 1.525 (1.393–1.670) |  |  |  |  |  | 1.121 (1.020–1.233) | 1.120 (1.019–1.231) |
| **Dietary diversity** |  |  |  |  |  |  |  |  |
| No | 1.000 |  |  |  |  |  |  | 1.000 |
| Yes | 0.818 (0.759–0.882) |  |  |  |  |  |  | 0.974 (0.899–1.055) |

Notes: Values are pooled odds ratios (ORs) with 95% confidence intervals from logistic regression models. Models were fitted separately within each country, and country-level log-odds estimates were pooled using random-effects meta-analysis. Abbreviations: CI, confidence interval; OR, odds ratio; WASH, water, sanitation, and hygiene.

| **Supplementary Table 3. Pooled KHB decomposition of the rural–urban difference in child stunting: sensitivity analysis restricted to children aged 24–59 months** | | | |
| --- | --- | --- | --- |
|  | **Pooled point estimate** | **95% confidence interval** | **% of total rural–urban association explained** |
| **Panel A. Explanatory factor groups examined separately** |  |  |  |
| **Distal socioeconomic resources** |  |  |  |
| **Total association** | 1.986 | 1.683–2.344 | NA |
| **Indirect association of each explanatory factor** |  |  |  |
| Household wealth | 1.250 | 1.19–1.313 | 32.5 |
| Maternal education | 1.073 | 1.037–1.111 | 10.3 |
| Maternal digital access | 1.201 | 1.145–1.259 | 26.7 |
| **Proximal household, care, and dietary conditions** |  |  |  |
| **Total association** | 1.894 | 1.622–2.211 | NA |
| **Indirect association of each explanatory factor** |  |  |  |
| Improved WASH components | 1.118 | 1.081–1.155 | 17.4 |
| Problems in accessing healthcare | 1.032 | 1.019–1.046 | 5 |
| Dietary diversity | 1.002 | 0.998–1.005 | 0.3 |
| **Panel B. Full decomposition model** |  |  |  |
| **All measured explanatory factors** |  |  |  |
| **Total association** | 2.008 | 1.698–2.374 | NA |
| **Indirect association of each explanatory factor** |  |  |  |
| Household wealth | 1.217 | 1.156–1.282 | 28.2 |
| Maternal education | 1.067 | 1.031–1.105 | 9.3 |
| Maternal digital access | 1.188 | 1.133–1.246 | 24.7 |
| Improved WASH components | 1.048 | 1.022–1.074 | 6.7 |
| Problems in accessing healthcare | 1.013 | 1.001–1.025 | 1.8 |
| Dietary diversity | 1.000 | 0.997–1.004 | 0 |

Notes: KHB decomposition was performed separately for each country using logistic regression models on the log-odds scale. Country-level KHB estimates were then pooled using random-effects meta-analysis. The pooled point estimates shown in the table are exponentiated pooled log-scale KHB estimates. The percentage of the total rural–urban association explained was calculated on the log-odds scale. For individual explanatory factors, it was calculated as the pooled log-scale contribution of that factor divided by the pooled log-scale total rural–urban association in the corresponding model, multiplied by 100. Panel A shows models in which the two groups of explanatory factors were examined separately: distal socioeconomic resources and proximal household, care, and dietary conditions. Panel B shows the full decomposition model in which all measured explanatory factors were examined together. Abbreviations: KHB, Karlson–Holm–Breen; WASH, water, sanitation, and hygiene; NA, not available.

| **Supplementary Table 4. Pooled odds ratios for childhood stunting from logistic regression models: complete-case sensitivity analysis** | | | | | | | | | |
| --- | --- | --- | --- | --- | --- | --- | --- | --- | --- |
| **Variable** | **Crude model** | **Model 1** | **Model 2** | **Model 3** | **Model 4** | **Model 5** | **Model 6** | **Model 7** |  |
|  |  | **Adjusted for child age, child sex, multiple birth status, birth order, and maternal age** | **Model 1 + household wealth** | **Model 2 + maternal education** | **Model 3 + maternal digital access** | **Model 4 + WASH conditions** | **Model 5 + healthcare access barriers** | **Model 6 + dietary diversity** |  |
| **Residence** |  |  |  |  |  |  |  |  |  |
| Urban | 1.000 | 1.000 | 1.000 | 1.000 | 1.000 | 1.000 | 1.000 | 1.000 |  |
| Rural | 1.777 (1.542–2.048) | 1.726 (1.505–1.979) | 1.275 (1.127–1.443) | 1.230 (1.088–1.391) | 1.175 (1.038–1.330) | 1.155 (1.018–1.310) | 1.144 (1.015–1.290) | 1.142 (1.013–1.288) |  |
| **Household wealth** |  |  |  |  |  |  |  |  |  |
| Poorest | 1.000 |  | 1.000 | 1.000 | 1.000 | 1.000 | 1.000 | 1.000 |  |
| Poorer | 0.848 (0.787–0.914) |  | 0.851 (0.792–0.915) | 0.862 (0.801–0.929) | 0.877 (0.815–0.943) | 0.883 (0.822–0.948) | 0.889 (0.827–0.957) | 0.889 (0.826–0.957) |  |
| Middle | 0.694 (0.633–0.761) |  | 0.730 (0.671–0.794) | 0.751 (0.689–0.818) | 0.780 (0.718–0.847) | 0.793 (0.726–0.865) | 0.802 (0.736–0.875) | 0.805 (0.738–0.877) |  |
| Richer | 0.587 (0.523–0.659) |  | 0.628 (0.556–0.709) | 0.654 (0.579–0.738) | 0.699 (0.621–0.787) | 0.723 (0.641–0.815) | 0.725 (0.642–0.819) | 0.727 (0.643–0.822) |  |
| Richest | 0.322 (0.260–0.399) |  | 0.391 (0.316–0.484) | 0.428 (0.346–0.528) | 0.504 (0.410–0.619) | 0.533 (0.438–0.648) | 0.537 (0.439–0.658) | 0.537 (0.439–0.658) |  |
| **Maternal education** |  |  |  |  |  |  |  |  |  |
| No education | 1.000 |  |  | 1.000 | 1.000 | 1.000 | 1.000 | 1.000 |  |
| Primary | 0.830 (0.745–0.925) |  |  | 0.900 (0.823–0.983) | 0.920 (0.844–1.003) | 0.927 (0.850–1.011) | 0.932 (0.853–1.017) | 0.933 (0.857–1.015) |  |
| Secondary or higher | 0.514 (0.429–0.616) |  |  | 0.721 (0.643–0.808) | 0.800 (0.716–0.894) | 0.814 (0.730–0.908) | 0.823 (0.736–0.921) | 0.822 (0.737–0.917) |  |
| **Maternal digital access** |  |  |  |  |  |  |  |  |  |
| Digitally excluded | 1.000 |  |  |  | 1.000 | 1.000 | 1.000 | 1.000 |  |
| Mobile phone only | 0.634 (0.577–0.696) |  |  |  | 0.795 (0.742–0.852) | 0.801 (0.748–0.858) | 0.802 (0.751–0.857) | 0.800 (0.749–0.854) |  |
| Less frequent internet use | 0.305 (0.224–0.414) |  |  |  | 0.537 (0.421–0.684) | 0.553 (0.438–0.698) | 0.555 (0.442–0.698) | 0.552 (0.439–0.693) |  |
| Regular internet use | 0.266 (0.192–0.369) |  |  |  | 0.502 (0.372–0.679) | 0.533 (0.401–0.710) | 0.541 (0.407–0.719) | 0.538 (0.407–0.711) |  |
| **Number of improved WASH facilities** |  |  |  |  |  |  |  |  |  |
| 0 | 1.000 |  |  |  |  | 1.000 | 1.000 | 1.000 |  |
| 1 | 0.798 (0.752–0.848) |  |  |  |  | 0.937 (0.881–0.996) | 0.941 (0.885–1.000) | 0.941 (0.885–1.000) |  |
| 2 | 0.589 (0.526–0.659) |  |  |  |  | 0.823 (0.755–0.897) | 0.835 (0.765–0.911) | 0.833 (0.764–0.907) |  |
| 3 | 0.349 (0.276–0.441) |  |  |  |  | 0.726 (0.612–0.863) | 0.738 (0.622–0.876) | 0.741 (0.628–0.874) |  |
| **Problems in accessing healthcare** |  |  |  |  |  |  |  |  |  |
| 0 | 1.000 |  |  |  |  |  | 1.000 | 1.000 |  |
| 1 | 1.192 (1.089–1.306) |  |  |  |  |  | 1.047 (0.976–1.124) | 1.049 (0.974–1.130) |  |
| 2 | 1.348 (1.192–1.525) |  |  |  |  |  | 1.069 (0.964–1.186) | 1.072 (0.966–1.189) |  |
| 3 | 1.402 (1.271–1.547) |  |  |  |  |  | 1.095 (1.007–1.191) | 1.099 (1.011–1.195) |  |
| 4 | 1.538 (1.328–1.781) |  |  |  |  |  | 1.157 (0.999–1.340) | 1.144 (0.995–1.315) |  |
| **Dietary diversity** |  |  |  |  |  |  |  |  |  |
| No | 1.000 |  |  |  |  |  |  | 1.000 |  |
| Yes | 0.812 (0.718–0.918) |  |  |  |  |  |  | 1.010 (0.912–1.118) |  |

Notes: Values are pooled odds ratios (ORs) with 95% confidence intervals from logistic regression models. Models were fitted separately within each country, and country-level log-odds estimates were pooled using random-effects meta-analysis. Abbreviations: CI, confidence interval; OR, odds ratio; WASH, water, sanitation, and hygiene.

| **Supplementary Table 5. Pooled KHB decomposition of the rural–urban difference in child stunting: complete-case sensitivity analysis** | | | | |
| --- | --- | --- | --- | --- |
|  | **Pooled point estimate** | **95% confidence interval** | **% of total rural–urban association explained** | |
| **Panel A. Explanatory factor groups examined separately** | |  |  | |
| **Distal socioeconomic resources** |  |  |  | |
| **Total association** | 1.823 | 1.574–2.111 | NA | |
| **Indirect association of each explanatory factor** |  |  | |  |
| Household wealth | 1.257 | 1.168–1.352 | 38.1 | |
| Maternal education | 1.050 | 1.023–1.079 | 8.2 | |
| Maternal digital access | 1.153 | 1.103–1.204 | 23.7 | |
| **Proximal household, care, and dietary conditions** |  |  |  | |
| **Total association** | 1.758 | 1.530–2.020 | NA | |
| **Indirect association of each explanatory factor** |  |  | |  |
| Improved WASH components | 1.103 | 1.066–1.142 | 17.4 | |
| Problems in accessing healthcare | 1.028 | 1.014–1.042 | 4.9 | |
| Dietary diversity | 1.006 | 0.996–1.016 | 1.1 | |
| **Panel B. Full decomposition model** |  |  |  | |
| **All measured explanatory factors** |  |  |  | |
| **Total association** | 1.836 | 1.584–2.128 | NA | |
| **Indirect association of each explanatory factor** |  |  | |  |
| Household wealth | 1.232 | 1.146–1.325 | 34.4 | |
| Maternal education | 1.043 | 1.017–1.069 | 6.9 | |
| Maternal digital access | 1.144 | 1.097–1.192 | 22.1 | |
| Improved WASH components | 1.046 | 1.024–1.069 | 7.4 | |
| Problems in accessing healthcare | 1.013 | 1.000–1.025 | 2.1 | |
| Dietary diversity | 1.000 | 0.990–1.010 | 0.0 | |

Notes: KHB decomposition was performed separately for each country using logistic regression models on the log-odds scale. Country-level KHB estimates were then pooled using random-effects meta-analysis. The pooled point estimates shown in the table are exponentiated pooled log-scale KHB estimates. The percentage of the total rural–urban association explained was calculated on the log-odds scale. For individual explanatory factors, it was calculated as the pooled log-scale contribution of that factor divided by the pooled log-scale total rural–urban association in the corresponding model, multiplied by 100. Panel A shows models in which the two groups of explanatory factors were examined separately: distal socioeconomic resources and proximal household, care, and dietary conditions. Panel B shows the full decomposition model in which all measured explanatory factors were examined together. Abbreviations: KHB, Karlson–Holm–Breen; WASH, water, sanitation, and hygiene; NA, not available.
